# 奥希替尼联合贝伐珠单抗在获得性*EGFR* T790M突变晚期非小细胞肺癌的疗效分析

**DOI:** 10.3779/j.issn.1009-3419.2022.101.56

**Published:** 2022-12-20

**Authors:** 艳斐 顾, 笑如 田, 若天 王, 小雪 李, 坤 钱, 元博 李, 靖颖 农

**Affiliations:** 1 100016 北京，北京和睦家医院启望肿瘤中心 New Hope Cancer Center, Beijing United Family Hospital, Beijing 100016, China; 2 100053 北京，首都医科大学宣武医院胸外科，国家老年疾病临床医学研究 Department of Thoracic Surgery, Xuanwu Hospital, Cancer Center of National Clinical Research Center for Geriatric Diseases, Capital Medical University, Beijing 100053, China

**Keywords:** 肺肿瘤, 表皮生长因子受体, 血管内皮生长因子, 奥希替尼, 贝伐珠单抗, Lung neoplasms, Epidermal growth factor receptor, Vascular endothelial growth factor, Osimertinib, Bevacizumab

## Abstract

**背景与目的:**

奥希替尼是对表皮生长因子受体（epidermal growth factor receptor, *EGFR*）T790M突变非小细胞肺癌（non-small cell lung cancer, NSCLC）有效的第三代靶向药物。尽管第一代EGFR酪氨酸激酶抑制剂（EGFR-tyrosine kinase inhibitors, EGFR-TKIs）联合贝伐珠单抗治疗可延长无进展生存期（progression-free survival, PFS），但奥希替尼联合贝伐珠单抗的后线疗效在IⅠ期研究WJOG8715L中并未得到肯定，目前该联合模式在中国人群中的数据仍非常有限。本研究旨在分析真实世界中奥希替尼联合贝伐珠单抗二线治疗的疗效，评价奥希替尼联合抗血管治疗模式在*EGFR* T790M获得性耐药突变NSCLC中的二线治疗价值。

**方法:**

收集2020年1月-2021年8月收治的第一、二代EGFR-TKIs治疗后伴EGFR T790M突变NSCLC患者共42例。16例接受二线奥希替尼联合贝伐珠单抗治疗，另26例接受二线奥希替尼单药治疗。分析患者的治疗效果。

**结果:**

联合组和单药组客观缓解率（objective response rate, ORR）相当（43.8% *vs* 50.0%, *P*=0.694）。两组中位PFS（14.0个月 *vs* 13.0个月，*P*=0.797）和总生存期（overall survival, OS）（29.0个月 *vs* 26.0个月，*P*=0.544）均未见统计学差异。*Cox*回归模型显示前线联合贝伐珠单抗治疗是奥希替尼后线单药或联合治疗PFS（*P*=0.045）及OS（*P*=0.023）更短的独立预测因素。

**结论:**

奥希替尼联合贝伐珠单抗二线治疗相比靶向单药治疗未见更好的疗效。

## Abstract (en)

**Background and objective:**

Osimertinib is a third-generation epidermal growth factor receptor-tyrosine kinase inhibitors (EGFR-TKIs) capable of overcoming non-small cell lung cancer (NSCLC) with *EGFR* T790M mutation. Although the addition of bevacizumab to 1^st^ generation EGFR-TKIs confers a significant improvement in progression-free survival (PFS) in treatment-naive EGFR mutant NSCLC patients, osimertinib plus bevacizumab combination failed to show prolongation in the phase 2 study WJOG8715L. Data of such combination in Chinese patients are still lacking. This study aimed to explore the efficacy of the addition of bevacizumab to osimertinib as second-line therapy in real-world data, and to evaluate the role of anti-angiogenesis plus osimertinib combination therapeutic strategies in pretreated Chinese NSCLC patients with acquired *EGFR* T790M mutation.

**Methods:**

A total of 42 advanced NSCLC patients with acquired *EGFR* T790M mutation after prior EGFR-TKIs treatment were collected between January 2020 to August 2021, with 16 cases treated with osimertinib plus bevacizumab and 26 cases treated with osimertinib. The treatment effect of patients were analyzed.

**Results:**

The objective response rate (ORR) in combination group and osimertinib group were 43.8% and 50.0% respectively (*P*=0.694). No statistically significant difference in median PFS (14.0 mon *vs* 13.0 mon, *P*=0.797) and overall survival (OS) (29.0 mon *vs* 26.0 mon, *P*=0.544) between the combination group and osimertinib group were observed. Prior history of bevacizumab was identified as an independent predictor of PFS (*P*=0.045) and OS (*P*=0.023).

**Conclusion:**

Our study demonstrated that adding bevacizumab to osimertinib could not show advantages in PFS and OS in pretreated NSCLC patients harboring *EGFR* T790M-mutation.

肺癌是全球癌症发病率第二位、死亡率第一位的恶性肿瘤^[1]^。非小细胞肺癌（non-small cell lung cancer, NSCLC）约占所有肺癌的85%，其中肺腺癌是最常见的组织病理学亚型，在NSCLC中的占比约55%。亚洲人群肺腺癌患者的表皮生长因子受体（epidermal growth factor receptor, *EGFR*）突变率高达51%^[2]^。尽管第一代和第二代EGFR酪氨酸激酶抑制剂（epidermal growth factor receptor-tyrosine kinase inhibitors, EGFR-TKIs）在*EGFR*敏感突变NSCLC中相比细胞毒化疗显示出更好的疗效^[3-7]^，但在10个月-13个月最终出现疾病进展，其中约50%的患者出现*EGFR*外显子20 T790M突变。第三代EGFR-TKIs甲磺酸奥希替尼（Osimertinib）在临床前及临床研究中无论对*EGFR*敏感突变外显子19 del和外显子21 L858R突变还是对*EGFR*外显子20 T790M突变均有良好疗效^[8, 9]^。在AURA3研究^[10]^中，奥希替尼后线治疗无进展生存期（progression-free survival, PFS）约为10个月，相比化疗更长，但仍然不能满足临床需求，因此有必要继续探索新的联合治疗策略。有基础研究^[11]^显示靶向治疗期间血管内皮生长因子（vascular endothelial growth factor, VEGF）表达水平会发生动态变化，EGFR-TKIs耐药通常伴VEGF水平升高。EGFR-TKIs联合VEGF抗体对*EGFR*突变肿瘤细胞系体现出协同抗肿瘤作用^[12, 13]^，临床前研究^[14]^也观察到奥希替尼联合贝伐珠单抗对*EGFR* T790M突变肿瘤的抑制作用。部分临床研究^[15-22]^显示第一代或第二代EGFR-TKIs联合抗血管药物具有较好疗效及安全性。本研究拟回顾性评估奥希替尼联合VEGF单抗贝伐珠单抗（Bevacizumab）在第一代或第二代EGFR-TKIs经治的晚期NSCLC中的疗效。

## 1 资料与方法

### 1.1 纳入与排除标准

① 纳入标准：经组织学病理证实的IV期NSCLC[国际肺癌研究协会第八版NSCLC肿瘤原发灶-淋巴结-转移（tumor-node-metastasis, TNM）临床分期]；*EGFR* 19del或*EGFR* L858R突变；既往曾接受第一代或第二代EGFR-TKIs治疗后出现影像学明确的疾病进展；进展后经组织或血液检测确认*EGFR* T790M突变；二线使用奥希替尼80 mg每日一次至少30 d，或奥希替尼80 mg每日一次联合贝伐珠单抗7.5 mg/kg每三周一次；至少具有一个可测量病灶。②排除标准：无组织学病理诊断；*EGFR*非经典突变；临床资料不完整；无可评价病灶；合并间质性肺病；具有较高出血或血栓风险；高血压未控制；伴脑膜转移；奥希替尼治疗中接受脑转移局部放射治疗。

### 1.2 临床资料

2020年1月-2021年8月，首都医科大学宣武医院胸外科及北京和睦家医院启望肿瘤中心共42例患者符合标准。临床资料包括患者性别、年龄、吸烟史、体力状况（performance status, PS）评分、EGFR突变亚型、是否伴TP53共突变、既往治疗、是否伴脑转移等。所有患者在治疗前完成血常规、肝肾功能、胸腹计算机断层扫描（computed tomography, CT）、头部磁共振成像（magnetic resonance imaging, MRI）及骨扫描或正电子发射型计算机断层显像（positron emission tomography-CT, PET-CT）检查，进行PS评分。用药治疗期间定期复查上述指标。服药1个月后进行影像学检查，随后每2个月进行疗效评价，直至出现疾病进展或不可耐受的毒副反应。

### 1.3 疗效评价

根据实体瘤治疗疗效评价标准（Response Evaluation Criteria in Solid Tumors, RECIST）1.1评价疗效。分为完全缓解（complete response, CR）、部分缓解（partial response, PR）、疾病稳定（stable disease, SD）和疾病进展（progressive disease, PD）。客观缓解率（objective response rate, ORR）包括CR和PR，疾病控制率（disease control rate, DCR）包括CR、PR和SD。PFS定义为从首次服药到客观证据证实疾病进展或任何原因引起死亡的时间。总生存（overall survival, OS）定义为从首次服药到肿瘤导致死亡时间。到截止时间时疾病未进展或死亡的患者以最后一次肿瘤随访评估的日期计算。与药物相关的毒性反应根据美国国立癌症研究所通用不良反应术语标准（National Cancer Institute Common Toxicity Criteria, NCI CTC）进行分级。

### 1.4 统计学方法

采用SPSS 23.0进行数据分析基线特征组间比较采用*Pearson*卡方检验或*Fisher*精确检验。生存分析采用*Kaplan-Meier*法，*Log-rank*检验组间差异。以*P* < 0.05为差异有统计学意义。通过*Cox*比例风险回归模型来探讨不同临床特征（脑转移、*EGFR*突变亚型、吸烟、*TP53*共突变）对于不同治疗中的PFS及OS获益情况，并在全组患者中探讨变量（前线是否联合贝伐、奥希替尼联合或单药、是否伴脑转移及*EGFR*突变亚型）是否会对PFS和OS产生独立影响。

## 2 结果

### 2.1 临床特征

自2020年1月-2021年8月纳入符合标准患者共42例，均为肺腺癌。全部患者均在一代或二代EGFR-TKIs治疗进展后通过肿瘤组织活检或外周血证实为*EGFR*外显子20 T790M突变阳性，基因检测手段为二代测序法。16例患者接受奥希替尼联合贝伐单抗治疗，另26例患者接受奥希替尼单药治疗。两组患者基本特征见表 1。其中脑转移者共11例，5例接受联合治疗，6例接受奥希替尼单药治疗，两个治疗组各有1例脑转移患者伴有中枢神经系统症状。

**表 1:** 全组患者基本特征[*n*（%）] The characteristics of patients [*n* (%)]

Characteristic		Osimertinib+bevacizumab (*n*=16)	Osimertinib (*n*=26)	*P*
Age, median (range) (yr)		64.5 (48-74)	62 (28-90)	
Gender	Male	5 (31.3)	6 (23.1)	0.559
	Female	11 (68.8)	20 (76.9)	
Smoker	No	11 (68.8)	22 (84.6)	0.265
	Yes	5 (31.3)	4 (15.4)	
ECOG performance status	< 2	14 (87.5)	20 (76.9)	0.688
	≥2	2 (12.5)	6 (23.1)	
*EGFR* mutation	*Exon* 20 T790M	16 (100)	26 (100)	0.808
	*Exon* 19 del	8 (50.0)	12 (46.2)	
	*Exon* 21 L858R	8 (50.0)	14 (53.8)	
Prior EGFR-TKIs	1^st^ Generation	14 (87.5)	24 (92.3)	0.628
	2^nd^ Generation	2 (12.5)	2 (7.7)	
*TP53* mutation	Yes	8 (50)	12 (46.2)	0.808
	No	8 (50)	14 (53.8)	
Prior bevacizumab	Yes	5 (31.3)	3 (11.5)	0.223
	No	11 (68.8)	23 (88.5)	
Brain metastasis	Yes	5 (31.3)	6 (23.1)	0.559
	No	11 (68.8)	20 (76.9)	
EGFR-TKIs: epidermal growth factor receptor-tyrosine kinase inhibitors; ECOG: Eastern Cooperative Oncology Group.				

### 2.2 疗效和生存

联合组和单药组ORR无统计学差异，分别为43.8%和50.0%（*P*=0.694），DCR均为100%。两组患者肿瘤缓解深度见图 1。随访截止时间至2022年5月31日。*Log-rank*检验显示联合组和单药组PFS无统计学差异，分别为14.0个月（95%CI: 3.739-24.261）和13.0个月（95%CI: 2.089-23.911）（*P*=0.797）（图 2A）。两组OS无统计学差异，分别为29.0个月（95%CI: 11.508-46.492）和26.0个月（95%CI: 22.657-29.343）（*P*=0.544）（图 2B）。*Cox*比例风险回归模型显示是否伴脑转移、是否吸烟、*EGFR*突变亚型、*TP53*共突变状态接受两种治疗模式的PFS（图 3A）和OS（图 3B）未见显著差异。在全组患者中，伴*TP53*共突变与不伴*TP53*共突变患者的PFS无统计学差异，分别为13.0个月（95%CI: 6.625-139.375）和20.0个月（95%CI: 7.046-32.954）（*P*=0.48）；OS分别为29个月（95%CI: 11.275-46.725）和24个月（95%CI: 8.794-39.206）（*P*=0.303）。共8例患者（单药组3例，联合组5例）在前线曾接受贝伐珠单抗治疗，前线曾接受与前线未接受过贝伐珠单抗治疗患者的中位PFS分别为9.0个月（95%CI: 4.974-13.026）和20.0个月（95%CI: 11.717-28.283）（*P*=0.017）（图 4A，图 4B）；中位OS分别为10.0个月（95%CI: 8.770-11.230）和26.0个月（95%CI: 21.343-30.657）（*P*=0.006, 9）（图 4C，图 4D）。*Cox*比例风险回归模型分析显示在包含了前线是否联合贝伐珠单抗、奥希替尼单药或联合治疗、是否伴脑转移及*EGFR*突变亚型的分析中前线曾联合贝伐珠单抗是PFS（*P*=0.045）和OS（*P*=0.023）更短的独立影响因素（表 2）。

**图 1:**
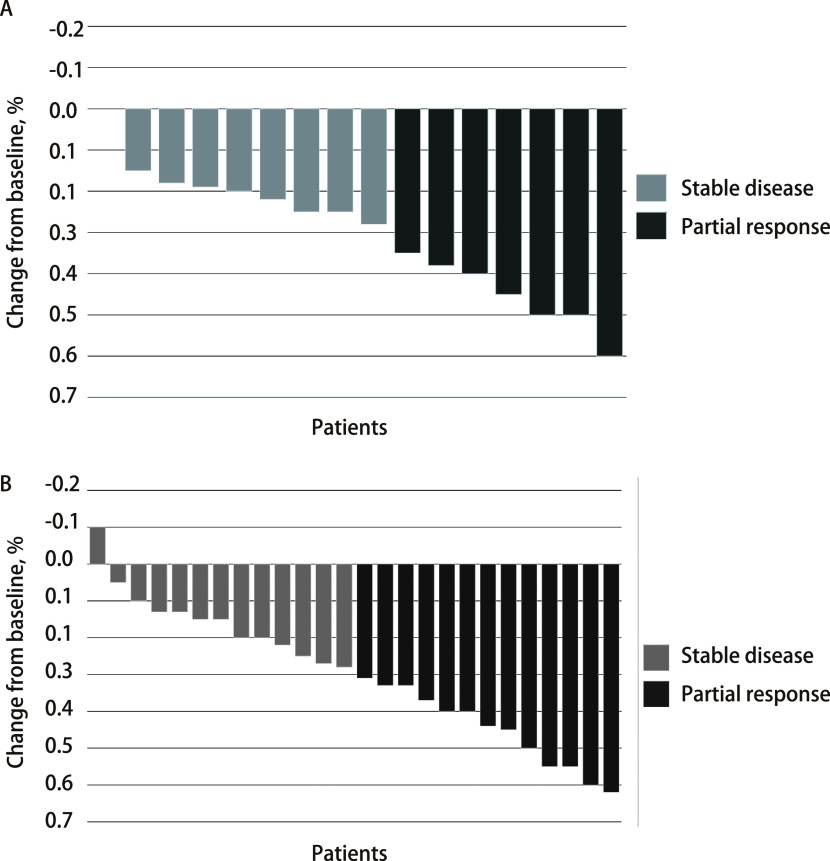
联合组及奥希替尼单药组靶病灶缓解深度。A：奥希替尼联合贝伐珠单抗组患者肿瘤靶病灶缓解深度；B：奥希替尼单药组患者肿瘤靶病灶缓解深度。 Response profiles of combination Osimertinib/bevacizumab group or Osimertinib group. A: Best overall response of the combination group Osimertinib/bevacizumab; B: Best overall response of the osimertinib group. The maximum percentage change in measurable tumor target lesions at the time of best response from baseline. (According to Response Evaluation Criteria in Solid Tumors version 1.1).

**图 2:**
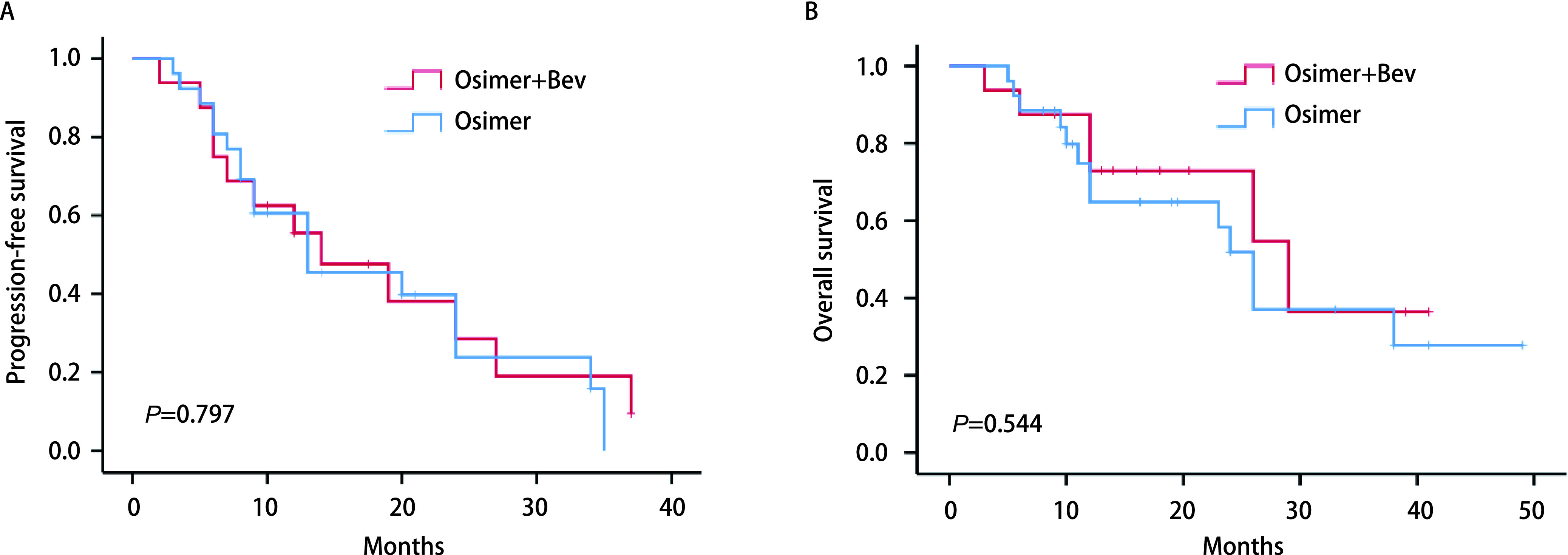
*Kaplan-Meier*法分析奥希替尼联合贝伐珠单抗组及奥希替尼单药组。A：无进展生存期；B：总生存期。 *Kaplan-Meier* curves of combination group and osimertinib group. A: Progression-free survival; B: Overall survival; Osimer: Osimertinib; Bev: Bevacizumab

**图 3:**
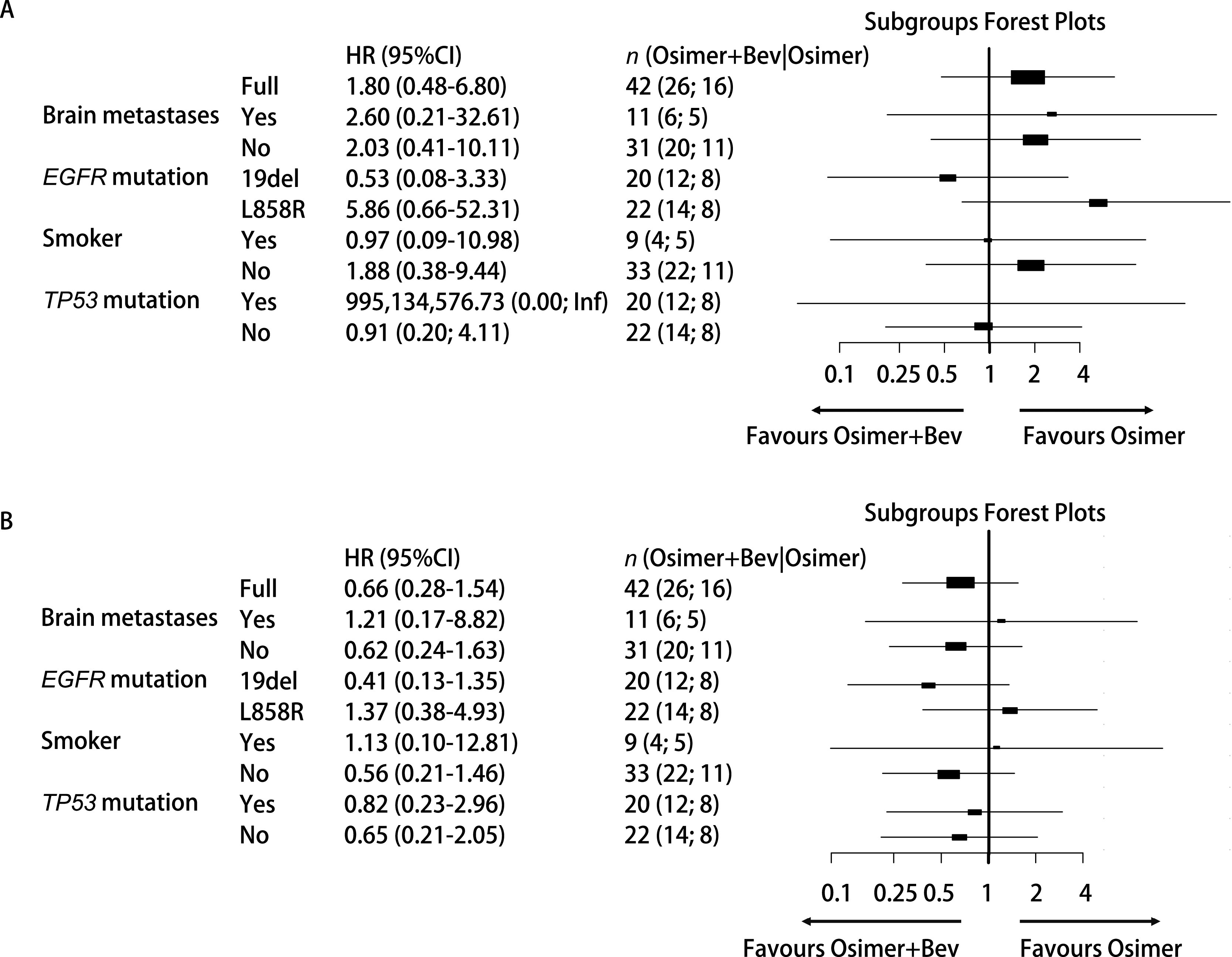
森林图显示各个亚组奥希替尼联合贝伐珠单抗或奥希替尼单药治疗的。A：无进展生存期获益；B：总生存期获益。 Forest plot for progression-free survival (A) and overall survival (B) within subgroups

**图 4:**
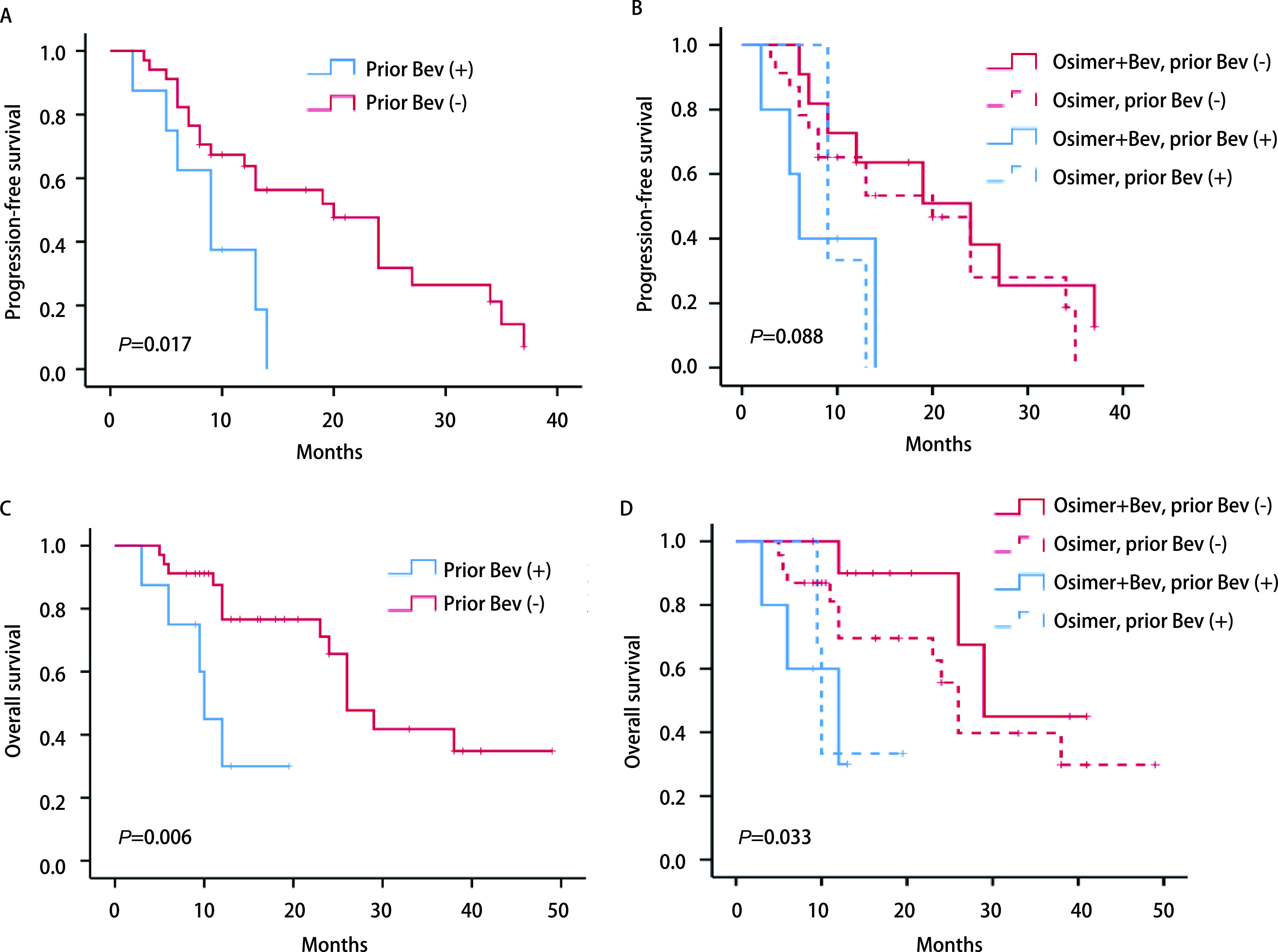
*Kaplan-Meier*法分析前线曾接受或未接受贝伐珠单抗治疗患者的无进展生存期和总生存期。A：前线曾接受或未接受贝伐珠单抗患者的无进展生存期；B：前线曾接受或未接受贝伐珠单抗患者分别进行奥希替尼联合贝伐珠单抗或奥希替尼单药治疗的无进展生存期；C：前线曾接受或未接受贝伐珠单抗患者的总生存期；D：前线曾接受或未接受贝伐珠单抗患者分别进行奥希替尼联合贝伐珠单抗或奥希替尼单药治疗的总生存期。 *Kaplan-Meier* curves of progression-free survival and overall survival in patients with or without prior bevacizumab treatment. A: *Kaplan-Meier* curves of progression-free survival in patients with or without prior bevacizumab treatment; B: Subgroup analysis of progression-free survival in combination group and osimertinib group according to prior bevacizumab treatment; C: *Kaplan-Meier* curves of overall survival in patients with and without prior bevacizumab treatment; D: Subgroup analysis of overall survival in combination group and osimertinib group according to prior bevacizumab treatment.

**表 2:** 无进展生存期与总生存期多因素*Cox*回归分析 *Cox* regression of progression-free survival and overall survival to assess the impact factor

	Progression-free survival			Overall survival	
	HR (95%CI)	*P*		HR (95%CI)	*P*
Prior bevacizumab (Yes *vs* No)	0.361 (0.133-0.979)	0.045		0.239 (0.069-0.819)	0.023
Treatment (Osimertinib+bevacizumab *vs* Osimertinib)	1.204 (0.558-2.596)	0.637		1.755 (0.623-4.944)	0.287
Brain metastases (Yes *vs* No)	0.886 (0.351-2.233)	0.797		0.686 (0.232-2.024)	0.495
EGFR mutation (19 del *vs* 21 L858R)	0.945 (0.452-1.976)	0.880		1.159 (0.454-2.961)	0.758

### 2.3 安全性

治疗后出现高血压及蛋白尿均发生在联合治疗组。其中高血压发生率37.5%（6/16），均为1级-2级；蛋白尿31.2%（5/16），均为1级-2级。常见不良反应还有腹泻和皮疹。腹泻均为1级-2级，在联合组和单药组发生率分别为37.5%（6/16）和38.5%（10/26）。皮疹均为1级-2级，在两组发生率分别为37.5%（6/16）和34.6%（9/26）。白细胞减低在两组发生率分别为18.8%（3/16）和15.4%（4/26）。

## 3 讨论

基础研究^[11-13]^及部分临床研究^[15-22]^数据显示一、二代EGFR-TKIs与贝伐珠单抗联合可提高疗效。FLAURA研究^[9, 23]^显示奥希替尼较第一代EGFR-TKIs吉非替尼具有更好疗效。因此对奥希替尼联合贝伐珠单抗是否带来更多临床获益寄予期望。基础研究^[14]^显示奥希替尼联合贝伐珠单抗能够延缓肿瘤进展，不增加肿瘤退缩幅度。在早期单臂临床研究^[24]^中也显示出初步疗效：49例初治*EGFR*敏感突变晚期肺癌接受奥希替尼联合贝伐珠单抗ORR达80%，中位PFS为18.4个月。然而，奥希替尼联合贝伐珠单抗对比奥希替尼单药在前线或后线治疗的前瞻性随机对照研究均未得出阳性结果。IⅠ期研究WJOG9717L^[25]^中，联合贝伐珠单抗（15 mg/kg）与奥希替尼单药一线治疗*EGFR*敏感突变的PFS分别为22.1个月和20.2个月（HR=0.862, 60%CI: 0.700-1.060; 95%CI: 0.531-1.397; *P*=0.213）。IⅠ期研究WJOG8715L^[26]^中奥希替尼加贝伐珠单抗（15 mg/kg）后线治疗*EGFR* T790M突变患者较奥希替尼单药未改善PFS（9.4个月*vs* 13.5个月，HR=1.44，80%CI：1.00-2.08，*P*=0.20）。另一项开放标签随机对照IⅠ期（ETOP10-16）BOOSTER研究^[27]^也得出相似结论。在155例获得性T790M突变患者的后线治疗中奥希替尼联合贝伐珠单抗组相比奥希替尼单药无论对PFS（15.4个月*vs* 12.3个月，*P*=0.83）还是OS（24.0个月vs 24.3个月，*P*=0.91）均无显著改善。在本研究中奥希替尼联合贝伐珠单抗后线治疗有效率相当，未见PFS延长，与WJOG8715L及BOOSTER研究结果相似。但是，另有研究显示某些特定场景下奥希替尼联合贝伐珠单抗可见生存改善。在一项回顾性研究^[28]^（*n*=96）中，奥希替尼治疗进展后如继续给予奥希替尼联合贝伐珠单抗（7.5 mg/kg）治疗相比化疗联合贝伐珠单抗可获得更长PFS（7个月*vs* 4.9个月，HR=0.415，95%CI：0.252-0.687，*P*=0.001），且奥希替尼联合贝伐珠单抗是OS更长的独立预测因子（HR=0.395, 95%CI: 0.225-0.692, *P*=0.001）。

脑转移是肺癌患者预后不良因素。*EGFR*敏感突变NSCLC脑转移发生率高达44%-63%^[29]^。有多中心回顾性研究^[30]^（*n*=149）显示联合贝伐珠单抗相比第一代EGFR-TKIs单药可延长脑转移患者的PFS、颅内FPS及OS。*Meta*分析^[31]^显示第一代TKIs联合放疗、联合贝伐珠单抗或第三代TKIs治疗均可改善脑转移患者生存。尽管第三代药物相比第一代药物具有更强的颅内肿瘤控制效力，但为提高疗效的多种尝试仍在开展。奥希替尼联合贝伐珠单抗对于脑转移是否可增效尚不清楚。前瞻性单臂二期研究^[32]^显示奥希替尼联合贝伐珠单抗模式在14例脑膜转移伴有*EGFR*敏感突变的NSCLC患者的颅内ORR达50%，中位PFS为9.3个月^[32]^。本研究并未观察到脑转移患者接受奥希替尼单药或奥希替尼联合贝伐珠单抗治疗的生存差异。与本研究结果类似，在WJOG9717L研究^[25]^亚组分析中基线伴脑转移患者两种治疗模式的PFS也未见差异。第三代EGFR-TKIs单药仍然是目前对于脑转移患者较有效的治疗方式^[33]^。共突变可能负向影响EGFR-TKIs疗效。研究^[34]^发现奥希替尼单药治疗前后合并*TP53*突变者的治疗中断时间更短。本研究中*TP53*共突变患者中位PFS在数值上短于无*TP53*共突变者，但两组未达到统计学差异。同时本研究结果中显示采取联合贝伐珠单抗治疗相比奥希替尼单药对于共突变患者并未见PFS延长。存在共突变的患者应探索更多治疗策略以期提升疗效。

本研究发现在全组患者中如前线治疗曾使用过贝伐珠单抗，在后线靶向治疗的PFS及OS较未使用过贝伐珠单抗患者更短，且是患者PFS及OS的独立预测因素，提示前线使用贝伐珠单抗可能对后线治疗产生负性影响。这一发现与WJOG8715L研究^[26]^相似。该研究中曾使用过贝伐珠单抗治疗患者的PFS明显短于未曾用过贝伐珠单抗的患者（4.6个月*vs* 11.1个月，HR=0.41，95%CI：0.13-1.27，*P*=0.03）。该人群接受单药或联合治疗的疗效是否存在差异，由于患者数量少（*n*=8），故未做统计学比较，有待继续积累样本分析。

奥希替尼联合贝伐珠单抗后线治疗NSCLC的疗效在中国人群中的数据非常有限。本研究中回顾分析了第一代或第二代EGFR-TKIs耐药后伴*EGFR* T790M阳性的晚期NSCLC后线接受奥希替尼联合贝伐珠单抗相比奥希替尼单药治疗未观察到ORR的提高和生存的延长。但本研究具有局限性如为回顾性小样本研究，亚组分析解读需较为谨慎。由于联合治疗相比靶向单药的不良反应率增加，因此如何精准找出明确能从靶向联合贝伐珠单抗治疗得到比单药更多获益的人群仍需更多探索。
